# The Use of Sanitary Latrines and the Practice of Open-Air Defecation in a Rural Setup in Perambalur District: A Cross-Sectional Study

**DOI:** 10.7759/cureus.32547

**Published:** 2022-12-15

**Authors:** Tamilarasan M, Maniprabhu S, Karthikeyan Kulothungan, Neethu George, Rock B Dharmaraj

**Affiliations:** 1 Community Medicine, Dhanalakshmi Srinivasan Medical College and Hospital, Perambalur, IND; 2 Community Medicine, K.A.P. Viswanatham Government Medical College, Trichy, IND

**Keywords:** human feces, sanitation, open defecation, rural population, household latrine

## Abstract

Background

Public health initiatives aim to decrease infectious diseases by enhancing sanitation, which is their primary goal. The practice of sporadically contaminating the environment with human feces has been around for generations and is embedded in the cultural behavior of villagers in India. This study aimed to estimate the proportion of people with access to latrine facilities and the proportion of people who practice open defecation in the villages of Perambalur, Tamil Nadu.

Methodology

This community-based, cross-sectional, analytical study was conducted in two rural villages in the Perambalur district for six months. After obtaining approval from the institutional ethics committee, we informed participants about the study’s purpose. We conducted the study in selected rural areas and included every single residence in the hamlet, irrespective of whether the residents were permanent or temporary. We did not include families that were not reachable at any point during the survey. A convenient sampling procedure was used to select 330 houses for the study. The lead investigator interviewed one individual from each household, preferably the head of the family. A semi-structured questionnaire was used to collect the pertinent information. All collected data were entered into Microsoft Excel (Microsoft Corp., Redmond, WA, USA), and SPSS software version 21 (IBM Corp., Armonk, NY, USA) was used to analyze the results.

Results

Only around 3.6% of the study participants lived in kutcha households, and about 99.1% of participants identified as Hindu. The proportion of household latrines used was 64.1% among the study participants. Of them, 52.3% engaged in open defecation. Only 4.7% of participants had access to an underground drainage system. Most participants gained knowledge from medical professionals (84.8%). Social media was the second most used source, accounting for about 60.7% of the total. The most frequent reason given for practicing open-air defecation was the perception that constructing restrooms would be expensive (76.3%), while the second most frequent reason was a lack of land (53.4%). An independent t-test found no statistically significant relationship between the availability of household latrines and the number of girls or boys, age, or family income. Compared to those living in semi-pucca and kutcha households, most participants (77.3%) who lived in pucca houses had household latrines. The chi-square test revealed that this proportional difference was statistically significant (p = 0.0001).

Conclusions

The study participants used household latrines 64.1% of the time. Of the participants, 52.3% engaged in open defecation. The government’s initiative to build toilets has only helped a quarter of the population, which needs to be improved. Only 5% of people living in rural areas have access to an underground drainage system. The results of our study provide a justification for the government program’s mandate that healthcare practitioners must deliver health education. Therefore, a personalized approach is required to overcome the behavioral barrier among rural people and achieve behavior change.

## Introduction

The primary goal of public health initiatives is to decrease infectious diseases by enhancing sanitation. Data on latrine use are the most crucial metric for evaluating how effectively sanitation programs are performing. According to 2018 data, there were 140 crore people in India, with 65% living in rural areas. Overall, 71.3% of the houses have access to bathrooms, with only 3.5% of having never used them. In Tamil Nadu, 16.2% of urban households and 73.33% of rural families practice open defecation [1].

Open-air defecation exacerbates diseases of the digestive tract [2]. It is also the biggest contributor to morbidity and mortality in low-income countries. Non-health implications of open-air defecation include a lack of privacy and safety for women and girls, decreased school attendance, and a lack of basic human dignity [3]. Almost 60% of open-air defecation cases worldwide occur in India [4].

Despite improvements in latrine accessibility between 2001 and 2011, over 50% of Indian homes still lack toilet facilities [5]. The Indian government provides financial aid to install toilets in connection with Information, Education & Communication activities. The Swachh Bharat Mission-Gramin (SBM-G) has accelerated the construction of rural restrooms in India [2].

According to the United Nations, open defecation must be eliminated by 2025. The Sustainable Development Goals include a goal to “achieve access to adequate sanitation for all and eradicate open defecation by 2030,” with a focus on women, girls, and those living in vulnerable situations [6].

Several tactics can be employed to increase toilet construction and promote its use globally. Sanitation marketing refers to actions taken to raise awareness and persuade households to adopt and use latrines [7]. Using the community-led total sanitation strategy, the neighborhood is organized to help it become an open defecation-free community [8,9]. By encouraging the use of latrines, school-led total sanitation seeks to alter the behavior of both students and communities [6].

The habit of randomly contaminating the environment with human feces is centuries old and firmly ingrained in the cultural behavior of villagers [10]. Awareness initiatives, media exposure, and pressure from students are some of the elements that are making individuals more conscious of the need for behavior change. Knowledge of the neighborhood and its citizens, the selection of pertinent messaging and technologies, and community involvement form the basis of a successful toilet marketing program [11]. We conducted this study in the villages of Perambalur, Tamil Nadu, considering the environment and intending to determine the proportion of people who have access to latrine facilities and the percentage of people who practice open defecation.

## Materials and methods

Study design

The present study was a community-focused, cross-sectional, analytical study.

Study population, place, and duration

We conducted this study over the course of six months in two rural communities in the Perambalur district. Each house in the village served as the study unit.

Ethical clearance and informed consent

Before the study began, we obtained an ethical clearance certificate from the Institutional Ethics Committee of Dhanalakshmi Srinivasan Medical College and Hospital (approval number: IECHS/ IRCHS/ No.152). Before consenting to participate in the study, we informed participants of the study’s goals.

Inclusion and exclusion criteria

We conducted the survey in selected rural areas and included every single dwelling with residents who were permanent or temporary residents of that hamlet. Families that could not be reached or contacted during the survey were not included in the study.

Sample size

According to a study conducted in 2019 by Kumar and Sinha et al., 79% of the rural population in the Perambalur district practiced open defecation [10]. The minimal sample size needed for this investigation was determined using the formula 4 × p × q/d^2^, where p stands for prevalence, q for the complement of p, and d for absolute error (precision), which was 5%. With a 95% confidence interval, 266 homes represented the minimum sample size needed for this study.

Data collection

For this study, we selected 329 households using a convenient sampling method from two villages in the Perambalur area. The lead investigator interviewed one individual from each household, preferably the head of the family. A semi-structured questionnaire was used to collect the pertinent information. The data tool asked questions about demographics, family information, information about their home latrines, and information about their open defecation practice.

Data entry and analysis

All collected data were entered into Microsoft Excel (Microsoft Corp., Redmond, WA, USA), and SPSS software version 21 (IBM Corp., Armonk, NY, USA) was used to analyze the results. We expressed all continuous data as the mean and standard deviation. We employed frequency and percentage to present the qualitative data similarly. To determine the relationship between the continuous variable and the outcome variable, the independent t-test was used (presence of open defecation). We also examined the relationship between the category variable and the outcome variable using the chi-square test. A P-value of less than 0.05 was considered statistically significant with a 95% confidence interval.

## Results

In this study, there were about 329 participants. The overall characteristics of the study participants are presented in Table 1. Only roughly 3.6% of the study participants lived in kutcha dwellings, and 99.1% of the participants identify as Hindu. The study participants used household latrines 64.1% of the time. Of them, 52.3% engaged in open defecation. Only 24% of the study participants benefited from the government program to build latrines.

**Table 1:** General characteristics of the study participants (n = 329).

Variables		Frequency (%)
Name of the village	Senjeri	205 (62.3%)
	Siruvachur	124 (37.7%)
Religion	Christian	3 (0.9%)
	Hindu	326 (99.1%)
Type of house	Kutcha	12 (3.6%)
	Pucca	164 (49.8%)
	Semi-pucca	153 (46.5%)
Availability of household latrine	No	118 (35.9%)
	Yes	211 (64.1%)
Open-air defecation	No	157 (47.7%)
	Yes	172 (52.3%)
Toilet availability	No toilet facility	118 (35.9%)
	Own toilet	205 (62.3%)
	Shared	6 (1.8%)
Benefitted from the government scheme to build a toilet	No	250 (76%)
	Yes	79 (24%)
Choice of spending money if given for any use	No idea	67 (20.4%)
	To build toilet	178 (54.1%)
	To buy household appliances	14 (4.3%)
	To renovate the house	70 (21.3%)

Table 2 provides a profile of the study participants who used the restroom at home. Overall, 94.8% of the participants used toilets in the Indian style (squatting). Approximately half of the participants (55%) flushed their toilets with their own bore water. Only 4.7% of the participants had access to an underground drainage system.

**Table 2:** Characteristics of the study participants who had household toilets (n = 211).

Variables		Frequency (%)
Type of latrine	Squatting	200 (94.8%)
	Western	11 (5.2%)
Place of toilet	Attached	157 (74.4%)
	Separated	48 (22.7%)
	Shared	6 (2.8%)
Source of water	Others	1 (0.5%)
	Own borewell	117 (55.5%)
	Public hand pump/tap	93 (44%)
Water supply	Continuous supply	148 (70.1%)
	Intermittent	63 (29.9%)
Sewage disposal	PIT	33 (15.6%)
	Septic tank	168 (79.6%)
	Underground disposal	10 (4.7%)
Provision of handwashing with soap nearby	No	38 (18%)
	Yes	173 (82%)

According to their source of health information, Table 3 lists the study participants who used public restrooms. Most of the participants acquired information from medical professionals (84.8%). Social media was the second-most used source, accounting for about 60.7% of the total.

**Table 3:** Description of the study participants who had household toilets according to their source of health information (n = 211). The source of health information was obtained as multiple-choice questions.

Source of health information		Frequency (%)
Healthcare providers	No	32 (15.2%)
	Yes	179 (84.8%)
Friends	No	112 (53.1%)
	Yes	99 (46.9%)
Family	No	94 (44.5%)
	Yes	117 (55.5%)
Social media	No	83 (39.3%)
	Yes	128 (60.7%)

Table 4 presents the study participants who did not have access to a toilet at home, along with their reasoning. The most frequent reason given was that they believed it would be expensive to install restrooms (76.3%), followed by a lack of land (53.4%).

**Table 4:** Information on the study participants’ reasons for not using a toilet in their homes (n = 118).

Variables		Frequency (%)
Lack of land availability	No	55 (46.6%)
	Yes	63 (53.4%)
Inadequate water supply	No	97 (82.2%)
	Yes	21 (17.8%)
Not aware of government schemes	No	92 (78%)
	Yes	26 (22%)
I feel it is not necessary	Yes	23 (19.5%)
	No	95 (80.5%)
Toilet associated with bad smell	No	112 (94.9%)
	Yes	6 (5.1%)
Toilet becomes a breeding place for flies	No	114 (96.6%)
	Yes	4 (3.4%)
Toilet should not be near the house	No	114 (96.6%)
	Yes	4 (3.4%)
Building toilet is costly	No	28 (23.7%)
	Yes	90 (76.3%)

Table 5 lists the characteristics of the study participants who practiced open defecation. About 59.2% of the participants did not regularly wash their hands with soap and water, and about 9.5% did not use slippers.

**Table 5:** Characteristics of the study participants with a habit of open defecation (n = 172).

Variables		Frequency (%)
Using slippers when going for open defecation	No	16 (9.3 %)
	Yes	156 (90.7%)
Handwashing after defecation with soap and water	No	93 (59.2%)
	Yes	64 (40.8%)

The relationship between predictor variables and the availability of restrooms is presented in Table 6. An independent t-test found no statistically significant relationship between the availability of household latrines and the number of girls or boys, age, or family income.

**Table 6:** Association between variables and availability of household latrines (n = 329).

Variables	Availability of latrine	Mean	Standard deviation	Mean difference	P-value
Number of females more than 60 years of age	No	0.246	0.4517	0.0088	0.872
	Yes	0.237	0.4887		
Number of males more than 60 years of age	No	0.237	0.4655	-0.0044	0.937
	Yes	0.242	0.5008		
Number of female members in the family	No	1.80	0.966	-0.208	0.071
	Yes	2.00	1.017		
Number of family members	No	3.746	1.5369	-0.2448	0.146
	Yes	3.991	1.4142		
Total family income per month (in INR)	No	8,364	3,713	-0.2192	0.156
	Yes	10,557	4,269		

The relationship between predictor variables such as religion, type of house, and availability of restrooms is displayed in Table 7. Compared to those living in semi-pucca and kutcha households, most study participants (77.3%) who lived in pucca houses had household latrines. The chi-square test revealed that this proportional difference was statistically significant (p = 0.0001).

**Table 7:** Association between variables and availability of household latrines (n = 329). *Fisher’s exact test was used.

Variables		Availability of household latrine		Chi-square vale	P-value
		No	Yes		
Religion	Christian	0 (0%)	3 (100%)	1.693*	0.555
	Hindu	118 (36.2%)	208 (63.8%)		
Type of house	Kutcha	6 (50%)	6 (50%)	25.172	<0.001
	Pucca	37 (22.6%)	127 (77.4%)		
	Semi-pucca	75 (49%)	78 (51%)		

## Discussion

The purpose of this study was to estimate the frequency of open-air defecation among rural residents and the existence of household latrines. According to our study, healthcare professionals were the primary source of health-related information regarding the importance of domestic toilets. This suggests a highly embedded primary healthcare delivery system in Tamil Nadu. However, only 24% of the survey participants who had bathrooms had benefited from government initiatives. The eligibility requirements specified in the funding system may be to blame for this. However, further research is required for this discussion.

Although there is a public healthcare system, our findings also revealed that 64.1% of households had latrines, and 52.3% engaged in open defecation. In addition, this study discovered that there was no correlation between family income, gender (across all ages), the number of family members, and the lack of latrines. This suggests that cultural practices remain common in rural regions even today.

According to a review by Behera et al. published in 2021, the Indian government launched the SBM-G in rural regions to provide everyone access to safe sanitation. Even after installing over one billion toilets in private homes, open defecation remains prevalent in rural regions at 52.1%. Our investigation also resulted in similar outcomes. The authors of the review pushed for a change in the attitudes of rural residents toward sanitation and restroom use. As a result, using restrooms should take precedence over constructing more restrooms [12].

Another study by Yogananth et al. found that 54.8% of people practiced open defecation, which is consistent with the findings of our study [1].

Routray et al. conducted a qualitative study in Odisha in 2015 using focus group discussions. Community members of all castes, genders, and age groups were given equal importance during the focus group discussion with non-governmental organization workers. The study concluded that simply improving infrastructure did not affect people’s behavior toward using sanitary latrines, and advised that behavioral impediments should be addressed through the program method [13].

Anuradha et al. conducted a cross-sectional study in Chennai, Tamil Nadu, involving 1,175 rural households. They discovered that 66.8% of people extensively used sanitary restrooms (household latrines: 62.5%; community latrines: 4.3%). The study also noted that 33.1% of people defecated in open fields [3]. The prevalence of open defecation among the rural population is concerning and calls for behavioral change among the people, even though their results diverged from our findings.

Veerapu et al. conducted a community-based interventional investigation among rural residents of Andhra Pradesh. Using health education materials about the knowledge, attitude, and practice (KAP) of using sanitary restrooms, hand washing, and wearing footwear, they conducted a study over a three-year period from 2012 to 2014. They concluded that there has been a statistically significant improvement in the population’s KAP [14]. Further research is necessary to see whether this understanding has caused any changes in behavior.

In the urban slums of eastern India, access to better sanitary facilities was practically universal, or 100%, according to research done in Kolkata by Kanungo et al. [15]. This disparity between urban slums and rural areas in the prevalence of sanitary toilet use suggests that the health education strategy and content should be adjusted to the needs of the local community.

Limitations

Because this was a quantitative study, it is impossible to discern how the rural population feels about open defecation and the use of sanitary latrines. The relationships described in the study need not be causative. A qualitative method can investigate this reasoning. We sampled only two villages via convenient sampling because of a lack of resources, which may impact how broadly the results may be applied.

## Conclusions

The study participants used household latrines 64.1% of the time. Of the study participants, 52.3% engaged in open defecation. It is necessary to increase the number of household latrines in rural areas. In India’s rural areas, open defecation is still widely practiced by over 50% of the population. The government’s toilet construction program, which now only benefits one-fourth of the population, needs to be expanded. Only 5% of people living in rural areas have access to an underground drainage system. Our study’s findings offered support for the government program’s requirement that healthcare practitioners provide health education. Therefore, a personalized approach is needed to overcome the behavioral barrier among rural people and achieve behavior change. To better understand the behavioral barrier among rural people, more qualitative research is required.

## References

[1] Yogananth N, Bhatnagar T (2018). Prevalence of open defecation among households with toilets and associated factors in rural south India: an analytical cross-sectional study. Trans R Soc Trop Med Hyg.

[2] Schmidt WP, Chauhan K, Bhavsar P (2020). Cluster-randomised trial to test the effect of a behaviour change intervention on toilet use in rural India: results and methodological considerations. BMC Public Health.

[3] Anuradha R, Dutta R, Raja JD, Lawrence D, Timsi J, Sivaprakasam P (2017). Role of community in Swachh Bharat Mission. Their knowledge, attitude and practices of sanitary latrine usage in rural areas, Tamil Nadu. Indian J Community Med.

[4] Bhardwaj A, Surana A, Mithra P, Singh A, Panesar S, Chikkara P (2013). A community based cross sectional study on use of sanitary latrines in a rural setup in Maharashtra. Healthline.

[5] Geetha K, Sampath Kumar S (2012). Open defecation: awareness & practices of rural districts of Tamil Nadu, India. Int J Sci Res.

[6] Bhatt N, Budhathoki SS, Lucero-Prisno DE (2019). What motivates open defecation? A qualitative study from a rural setting in Nepal. PLoS One.

[7] Jenkins MW, Scott B (2007). Behavioral indicators of household decision-making and demand for sanitation and potential gains from social marketing in Ghana. Soc Sci Med.

[8] Crocker J, Saywell D, Bartram J (2017). Sustainability of community-led total sanitation outcomes: evidence from Ethiopia and Ghana. Int J Hyg Environ Health.

[9] Harter M, Mosch S, Mosler HJ (2018). How does community-led total sanitation (CLTS) affect latrine ownership? A quantitative case study from Mozambique. BMC Public Health.

[10] Kumar R, Sinha SP (2019). Socio-cultural determinants of open defecation in rural households of Perambalur district, Tamil Nadu. Int J Community Med Public Health.

[11] Mcconville J, Candidate MS (2003). How to Promote the Use of Latrines in Developing Countries. https://www.ircwash.org/sites/default/files/Conville-2003-How.pdf.

[12] Behera MR, Pradhan HS, Behera D, Jena D, Satpathy SK (2021). Achievements and challenges of India's sanitation campaign under clean India mission: a commentary. J Educ Health Promot.

[13] Routray P, Schmidt WP, Boisson S, Clasen T, Jenkins MW (2015). Socio-cultural and behavioural factors constraining latrine adoption in rural coastal Odisha: an exploratory qualitative study. BMC Public Health.

[14] Veerapu N, Subramaniyan P, Praveenkumar BA, Arun G (2016). Promotion of sanitation and hygiene in a rural area of South India: a community-based study. J Family Med Prim Care.

[15] Kanungo S, Chatterjee P, Saha J, Pan T, Chakrabarty ND, Dutta S (2021). Water, sanitation, and hygiene practices in urban slums of eastern India. J Infect Dis.

